# Dietary Pattern and Its Association with the Prevalence of Obesity, Hypertension and Other Cardiovascular Risk Factors among Chinese Older Adults

**DOI:** 10.3390/ijerph110403956

**Published:** 2014-04-10

**Authors:** Jing Sun, Nicholas J. Buys, Andrew P. Hills

**Affiliations:** 1 Griffith Health Institute, Griffith University, Gold Coast Campus, Parkland Q4222, Australia; E-Mails: n.buys@griffith.edu.au (N.J.B.); ahills@mmri.mater.org.au (A.P.H.); 2 School of Medicine, Griffith University, Gold Coast Campus, Parkland Q4222, Australia; 3 Mater Mothers’ Hospital, South Brisbane, Queensland 4101, Australia; 4 Centre for Nutrition and Exercise, Mater Research Institute, University of Queensland, South Brisbane, QLD 4101, Australia

**Keywords:** dietary pattern, obesity, hypertension, metabolic syndrome, older adults

## Abstract

*Aim*: This article examined the association between dietary patterns and cardiovascular risk factors in Chinese older adults. *Methods*: For this study, older adults with one or more cardiovascular risk factors or a history of cardiovascular disease were randomly selected using health check medical records from the Changshu and Beijing Fangshan Centers for Disease Control and Prevention. Exploratory factor analysis and cluster analysis was used to extract dietary pattern factors. Log binomial regression analysis was used to analyse the association between dietary patterns and chronic disease related risk factors. *Results*: Four factors were found through factor analysis. A high level of internal consistency was obtained, with a high Cronbach’s alpha coefficient of 0.83. Cluster analysis identified three dietary patterns: healthy diet, Western diet, and balanced diet. Findings in this sample of Chinese adults correspond to those reported in previous studies, indicating that a Western diet is significantly related to likelihood of having obesity, hypertension and the metabolic syndrome. The identification of distinct dietary patterns among Chinese older adults and the nutritional status of people with chronic diseases suggest that the three dietary patterns have a reasonable level of discriminant validity. *Conclusions*: This study provides evidence that a FFQ is a valid and reliable tool to assess the dietary patterns of individuals with chronic diseases in small- to medium-size urban and rural settings in China. It also validates the significant association between dietary pattern and cardiovascular disease risk factors, including body mass index, blood pressure, triglycerides, and metabolic conditions. Clinical diagnosis of chronic disease further confirmed this relationship in Chinese older adults.

## 1. Introduction

Examining the relationship between dietary patterns in Chinese older adults with chronic disease is of particular importance given the increasing population and prevalence of age-related chronic disease. Chinese adults over 50 years of age have a higher prevalence of hypertension, obesity, diabetes, cardiovascular disease, stroke and bodily pain than do people in younger age groups [1,2]. Insufficient or unhealthy nutritional intake may cause this group to have an increased risk of morbidity and mortality [3]. 

The Food Frequency Questionnaire (FFQ) is commonly used to assess dietary patterns associated with clusters of frequently consumed food items [4,5,6]. A number of such questionnaires have been developed and widely used in Western countries with the main food patterns identified as vegetable-based, Western, and sweet- and fat-dominated patterns [7]. The vegetable-based or healthy food pattern comprises foods rich in milk, fruits, vegetables, grains, poultry, fish and nuts [8,9]. The Western pattern comprises foods rich in red meats and fats, and the sweet- and fat-dominated food patterns include sweets and added fat through consumption of sugary drinks, fast and processed food [8,9]. 

Associations have been found between dietary patterns and health outcomes and biomarkers, including the body mass index (BMI), serum cholesterol and blood pressure (BP) [4]. The vegetable-based dietary pattern is significantly related to a reduced likelihood of having metabolic syndrome, while consumption of Western and sweet- and fat-dominated dietary pattern foods is associated with a high risk of having this condition [10]. Previous studies of dietary patterns suggest that adherence to a healthy food pattern may protect against metabolic syndrome [4,10], risk of cardiovascular disease, obesity and hypertension. Dietary patterns often consist of a number of food items and there is no single food item that contributes to these risk factors [4,10]. This suggests that an overall healthy food pattern, rather than the consumption of specific food items, protects against chronic disease–related risks, such as high BMI, high waist-hip ratio, glucose intolerance and a high level of lipoprotein [11]. Therefore, examining dietary patterns and the potentially interactive effects among nutrients that might affect health outcomes, may be more appropriate in determining the relationship between dietary pattern and chronic disease-related risk factor than identifying consumption of specific food items [4,12,13]. 

Examination of the relationship between dietary patterns and likelihood of obesity and chronic disease risk for Chinese older adults is important to provide specific dietary information to inform tailored interventions to treat and reduce the risk of multiple diseases in this population [14,15]. To date, most studies of the dietary patterns of older people have been based on Western populations. However, the questionnaires used in these studies may not be suitable to assess the nutritional inadequacy of diets among the rapidly growing ageing population in China, many of whom reside in small- or medium-size cities and adjacent rural areas, where traditional Chinese foods, rather than Western foods, dominate dietary patterns. The purpose of this study therefore was to assess nutritional status in older adults with chronic diseases resident in small- to medium-sized cities in China using an instrument validated on the population in question. 

## 2. Methods

A cross-sectional study design was used with data collected in August 2012. Participants consisted of adults aged 50 years and above, located in two community environments in Changshu City, Jiangsu Province, southern China, and Beijing Fangshan District, Beijing, northern China. Changshu City was chosen because it is a typical small- to medium-sized city located in southern China, and therefore it represents southern food patterns. Beijing Fangshan District was chosen because its mixture of urban and regional areas making it typical of northern China in terms of food consumption. 

Changshu CDC and Beijing Fangshan CDC randomly selected 1,219 people of at least 50 years of age, registered for health checks in their medical records for cardiovascular related risk factors, to participate in the study. Of these, 1,070 (310 males and 760 females) gave written informed consent to participate, including 744 adults in Changshu and 326 adults in Beijing, representing an overall response rate of 88%. Participants then completed a Food Frequency Questionnaire, an anthropometry assessment, and biomedical measurements. A face-to-face interview was conducted by trained researchers either at the Beijing Fangshan CDC and Changshu CDC affiliated community health centres to ensure all participants met the inclusion criteria regarding age and medical status. Inclusion criteria included people who were aged 50 years and older, the presence of one or more cardiovascular related risk factors, *i.e.*, abnormal level blood pressure, abnormal BMI or waist circumference, and abnormal glucose or cholesterol or lipid protein level based on International Diabetes Federation standard [16]. People who had diagnosed metabolic syndrome or cardiovascular diseases or their related risk factors but were under medication at the time were also eligible to participate in the study. People who could not provide written consent to the study, or who had neurological impairments, and/ or severe mental illness were excluded from the study. 

Anthropometry assessment involved height and weight measurements, and biomedical measurements included cholesterol and blood glucose measurements taken by qualified physicians. These are described below. Approval to conduct the study was obtained from the Beijing Fangshan CDC and Changshu CDC.

## 3. Measurements

Food Frequency Questionnaire (FFQ): A FFQ specific to the Chinese context was developed during this study using the following process. The 24-h dietary recall method was used to collect participants’ food intake over two week days and one weekend day. Pictures of 50 common foods, in typical Chinese measures (one Liang equals 50 g) were provided, and participants were asked to indicate the amount they had consumed and how often they had consumed them (daily, weekly or monthly). Results indicated 34 frequently consumed items, grouped as below: rice, flour, grains, beans, soybeans, soybean products, nuts including peanuts and sunflower seeds, dark-coloured vegetables, light-coloured vegetables, edible fungi and algae (such as mushrooms, agaric and kelp), fruits, pork, poultry, seafood, beef/lamb/other red meat, organ meat, processed meat (such as ham, luncheon meat, canned meats), eggs, milk and yogurt, milk powder and cheese, confectionery, fast food, sugar, puffed food and pastries. The amount of each food item consumed was calculated as per day per person. 

The 34 food items, representing 90% of commonly consumed foods in the two cities, were then used in the FFQ. For each food item on the questionnaire, participants were asked to select the frequency of intake (daily, weekly, monthly, yearly or never) and indicate the amount of consumption in ‘units’ (with one unit equaling 50 grams). Frequency of intake was converted to the number of intakes per day. Of the 1,070 questionnaires completed, 1,064 were used in the study. Six of the questionnaires were not included because more than 50% of the questions were incomplete. Individual questions with missing items were imputed by multiple imputation methods with five imputations. 

Anthropometry and biomedical measurements: BMI, waist and hip girth (cm), and systolic and diastolic blood pressure (SBP and DBP) were measured to provide concurrent validity for the FFQ and to assess chronic disease–related risk factors when correlated with anthropometry measures. BMI, an indicator of relative weight was calculated using measured body weight and height. In Chinese adults, a BMI below 19 kg/m^2^ is considered underweight, while a BMI higher than 28 kg/m^2^ is considered obese. Waist and hip girth (cm) were measured with an anthropometric tape over light clothing. Waist girth was measured at the minimum circumference between the iliac crest and the ribcage, and hip girth at the maximum width over the greater trochanters. The waist-to-hip ratio was then calculated as waist divided by hip measurements. Abdominal obesity was defined based on waist circumference and the World Diabetes Federation standard [17]. SBP and DBP were measured using an inflatable cuff wrapped around the upper arm and attached to an electronic monitor that gave a digital readout of the BP (mmHg) and pulse. The criteria for elevated BP are SBP > 135 mmHg and DBP > 85 mmHg, based on the International Diabetes Federation (IDF) and China CDC criteria [17]. Blood glucose and total cholesterol were measured to assess the concurrent validity of the FFQ relating to the correlation between diet and biomedical measures. According to the IDF definition [16,17], abnormal blood glucose is ≥5.6 mmol/L and an abnormal cholesterol level is total cholesterol ≥5.17 mmol/L.

Chronic disease: Chronic disease data were collected from both Changshu CDC and Fangshan CDC medical records (8). As part of the FFQ, data regarding chronic disease condition, including diabetes, heart disease, hypertension, stroke and bodily pain, using a yes or no scale were collected, to assess discriminant validity for the FFQ. 

Statistical analysis: Exploratory factor analysis was used to assess the FFQ’s psychometric properties. Dietary patterns were derived using factor analysis with factor loadings that were extracted with the principal component method and Varimax rotation, using 34 food items from the FFQ. The frequency of consumption of the 34 food items was converted to weekly equivalent food consumption frequencies, and these were entered into the factor analysis. The majority of items were retained because they appeared on the FFQ to reduce the number of subjective decisions made in determining dietary patterns. This was done based on previous research that recommends this approach. Frequency variables were log-transformed to improve normality prior to the factor analysis. The Kaiser-Meyer-Olkin (KMO) to measure sampling adequacy and Bartlett’s test of sphericity to assess the adequacy of test items and sample size for factor analysis were applied [18]. High values (>0.50) in the KMO suggest that the study had adequate sample size. Factor analysis using Varimax rotation methods was used to extract the potential underlying dietary intake factors in older people. An eigenvalue of >1.00 was used to determine the number of dietary intake factors. A Scree Test and interpretability of the results were used to reduce the meaning redundancy of the factors. Finally, items with factor loadings >0.30 were considered as belonging to a factor (16). 

The reliability and validity of the instruments were assessed to evaluate the psychometric properties of the FFQ. Cronbach’s alpha coefficient was used to determine the internal consistency of each extracted factor [19]. Construct validity analysis was conducted to examine whether the items correlated with the extracted construct, and to calculate the amount of variance explained by the items. Corrected item-total correlations between the item score and the scores obtained from all extracted constructs was used to indicate the level of convergent validity, with higher correlations associated with higher levels of convergent validity on each item. The lower the correlation of an item’s score with the scores of the other constructs, the higher the level of discriminant validity of that item [20]. Following factor analysis, cluster analysis was used to identify the dietary pattern. The differences among dietary patterns in macronutrients, micronutrient and biomedical factors were analysed by general linear model when confounding factors including age, gender were controlled in the analysis. The log binomial regression model and the log-linear poisson approximation was used to analyse the association between dietary patterns and the indices of chronic diseases, including SBP, DBP, BMI and waist circumference, hip circumference, waist-hip ratio, serum cholesterol, HDL, triglycerides, LDL, and fasting glucose. For each risk factor, the risk range is the range of fitted risk probabilities across all combinations of the other confounding factors. The 95% CIs are based on 1,000 bootstrap replications, for which both methods are always converged. Confounding factors including education, employment, income and exercise level were controlled in in relation to the association between dietary patterns and prevalence of chronic diseases. 

## 4. Results

The overall KMO measure for the 34 items initially analysed was 0.826—much higher than the cut-off point of 0.5, thus supporting the adequacy of the sample size used in the factor analysis. The correlation coefficients of all items were more than 0.50, indicating that all items fit well with each other. Bartlett’s test of sphericity verified that the inter-item correlations were sufficient (chi square = 6.66; df = 561; *p* < 0.001). Factor analysis revealed four unique dietary patterns (see Table 1). The dietary factor for males and females were similar in relation to the number of factors identified. Due to the similar pattern revealed between males and females, these groups were combined in the analysis. 

The four unique dietary patterns identified were labelled as a traditional food factor, western food factor, a processed and fast food factor, and animal organs factor. The traditional food factor consisted of fish and prawn, fruit, vegetables, pork, rice, poultry, and bean products. The Western food factor consisted of red meat, flour, light vegetable, grains, beans, soybeans, potato, peanuts and sunflowers, fresh milk. The fast and processed food factor including fast food, puffed foods, meat snacks, carbonated drinks, cheese, milk drinks, confectionery and cookies and pastries, and vegetable and fruits products. The animal organ factor included animal livers, animal blood, and cheese. The four factors explained 34% of total variance, with the traditional food factor comprising 15.16%, western food factor, 8.66%, fast and processed food factor, 5.92%, and animal organ foods factor, 4.80% of the variance, respectively. One food item, milk powder, was excluded from the factor analysis due to its very low frequency of consumption and low factor loading. 

**Table 1:** Factor analysis results.

Food Items	Factor 1	Factor 2	Factor 3	Factor 4
1. Fish and prawn	**0.664**	-0.011	0.171	-0.274
2. Rice	**0.651**	-0.168	0.006	0.144
3. Eggs	**0.573**	0.303	0.01	-0.039
4. Pork	**0.555**	0.263	0.121	-0.18
5. Dark vegetable	**0.530**	0.285	-0.053	0.215
6. Yogurt	**0.481**	0.028	0.013	0.147
7. Cookie pastries	**0.459**	0.126	0.334	-0.34
8. Fungus	**0.458**	0.236	0.074	0.385
9. Apple pear Banana and Strawberry	**0.402**	0.363	-0.043	0.197
10. Poultry	**0.385**	0.132	0.273	0.08
11. Bean products	**0.320**	0.131	0.072	0.082
12. Milk powder	0.140	0.108	0.103	0.103
13. Flour	-0.122	**0.695**	-0.032	-0.020
14. Light vegetable	0.037	**0.604**	-0.004	0.159
15. Grains	0.105	**0.596**	0.052	-0.329
16. Beans	0.185	**0.576**	0.073	-0.300
17. Soybeans	0.145	**0.541**	0.019	0.023
18. Potato	0.136	**0.437**	0.094	0.021
19. Water	0.271	**0.389**	-0.087	0.195
20. Peanuts sunflower	0.190	**0.373**	0.15	0.087
21. Fresh milk	0.070	**0.363**	0.024	0.209
22. Red meats	0.106	**0.355**	0.208	0.182
23 Fruit vegetable products	0.121	0.03	**0.652**	-0.037
24. Puffed food	0	0.043	**0.569**	0.066
25. Confectionery	0.169	-0.026	**0.562**	0.002
26. Carbonated drinks	-0.039	0.047	**0.513**	0.004
27. Fast food	0.059	-0.022	**0.51**	0.132
28. Commercial fruit vegetable juice	-0.038	0.072	**0.497**	0.291
29. Meat snack	0.143	-0.005	**0.488**	-0.016
30. Milk drink	-0.029	0.136	**0.456**	0.328
31. Fresh fruit juice	0.05	0.099	**0.417**	0.228
32. Animal liver	0.117	0.087	0.300	**0.517**
33. Animal blood	0.042	0.116	0.304	**0.469**
34. Cheese	0.103	-0.011	0.085	**0.398**
Eigen value	5.16	2.95	2.01	1.63
Percentage of variances (%) explained	15.16%	8.66%	5.92%	4.80%

Note: Figures in bold indicate factor loading is more than cut-off score of 0.30 and is considered to belong to the corresponding dimension in the column.

Cronbach’s alpha indicated that there was a high inter-item reliability (0.83) for the new FFQ questionnaire. The Spearman correlation coefficients between the two food pattern scores were in the range of 0.25 to 0.38, suggesting that these are four discrete and independent food factor. The item—total correlations ranged from 0.12 to 0.50 (*p* < 0.05), indicating that each item contributed to the total score. The reliability scores for 33 food items was high, with a Cronbach’s alpha coefficient of 0.76. 

A cluster analysis based on the four factors further identified three clusters that respectively accounted for 42.8% (n = 431), 36.3% (n = 365), and 20.9% (n = 210) of the total sample. These clusters were labelled as the healthy dietary pattern, the Western dietary pattern, and the balanced dietary pattern (see Table 2). Adults with a healthy dietary pattern (6,858.42 KJ) and the Western dietary pattern (6,320.45 kJ), had significantly higher total energy intake than those (3,184.03 KJ) with the balanced dietary pattern (both *p* < 0.001) (see Table 4).

**Table 2:** Classification of subjects by cluster analysis using factor score.

Food Patterns	Cluster 1 a (n = 431)	Cluster 2 b (n = 365)	Cluster 3 c (n = 210)	F	*p*	*Post-hoc*
Traditional Food	885.50 (327.32)	766.44 (349.01)	356.49 (261.86)	192.476	<0.001	A > B ***
						B > C ***
						A > C ***
Western Food	782.16 (283.25)	1683.21 (418.76)	148.69 (183.81)	1629.666	<0.001	B > A ***
						B > C ***
						A > C ***
Fast Food	48.45 (125.38)	46.81 (102.01)	15.76 (63.59)	7.483	<0.001	C < A **
						C < B **
Animal Organs	3.61 (10.79)	3.68 (10.87)	1.28 (7.46)	4.422	<0.01	C < A **
						C < B **

Notes: Statistical significance: ** *p* < 0.01, *** *p* < 0.001.

The characteristics of adults according to the dietary patterns are shown in Table 3. Adults with the healthy dietary pattern tended to have higher socioeconomic status than those with western dietary pattern and balanced dietary pattern. 

**Table 3:** Dietary patterns and characteristics of the participants.

Demographic Factors	Healthy Dietary	Western Food Dietary	Balanced Dietary	χ^2^	*p*
**Age**					
50–64 years	224 (66.7%)	102 (71.3)	127 (66.8)	1.09	0.58
≥65 years	112 (33.3)	41 (28.7)	63 (33.2)		
**Gender**					
Female	342 (79.4)	301 (82.5)	161 (80.3)	1.26	0.53
Male	89 (20.6)	64 (17.5)	41 (19.7)		
**Education**					
<10 years	203 (47.4)	251 (69.3)	97 (47.1)	**46.80**	**<0.001**
12 years	159 (37.1)	77 (21.3)	83 (40.3)		
≥Bachelor	66 (15.4)	34 (9.4)	26 (12.6)		
**Employment**					
Retired	344 (79.8)	171 (46.8)	188 (90.4)	**154.13**	**<0.001**
Working	87 (20.2)	194 (53.2)	20 (9.6)		
**Income**					
<20,000 yuan	219 (51.5)	273 (75.6)	104 (51.2)	**63.99**	**<0.001**
20,000–39,999 yuan	128 (30.1)	57 (15.8)	76 (37.4)		
>40,000 yuan	78 (18.4)	31 (8.6)	23 (11.3)		
**Marital Status**					
Married	380 (88.2)	324(88.8)	179 (86.1)	2.554	0.64
Widowed	36 (8.4)	31 (8.5)	18 (8.7)		
Never Married	15 (3.5)	10 (2.7)	11 (5.3)		

Note: Figures in bold show statistical significance at *p* value less than 0.05.

**Table 4:** Association between dietary patterns and nutrients.

Nutrients and Energy Intake	Healthy Dietary (n = 430) M(SD)	Western Dietary (n = 365) M(SD)	Balanced (n = 160) M(SD)	F	*p*
**Macro Nutrients**					
Total Energy (KJ) *	6,858.42 (1,551.43) **^a^**	6,320.45(2,943.82) **^a^**	3,184.03 (1,478.19) **^b^**	7.23	<0.001
Protein (g) *	92.81 (139.01) **^a^**	87.55 (44.00) **^a^**	37.23 (23.83) **^b^**	20.01	<0.001
Fat (g) *	76.75 (317.01) **^a^**	67.651 (48.18) **^a^**	26.63 (18.63) **^b^**	3.22	0.04
Saturated fat (g) *	16.98 (47.55) **^a^**	14.89 (9.13) **^a^**	5.82 (4.61) **^b^**	6.98	<0.001
Poly unsaturated fat (g) *	21.40 (90.29) **^a^**	18.37 (14.41) **^a^**	7.15 (5.98) **^b^**	3.17	0.04
Mono unsaturated fat (g) *	33.45 (165.84) **^a^**	29.83 (24.13) **^a^**	11.82 (8.04) **^b^**	2.21	0.11
Cholesterol (mg) *	289.40 (157.09) **^a^**	**291.29 (183.20) **^b^****	140.64 (160.63) **^c^**	52.77	<0.001
Carbohydrate (g) *	128.91 (71.88) **^a^**	122.06 (46.88) **^a^**	86.59 (36.37) **^b^**	31.52	<0.001
Sugars (g) *	33.37 (33.70) **^a^**	33.39 (22.23) **^a^**	11.48 (12.17) **^b^**	44.05	<0.001
Starch (g) *	94.43 (42.93) **^a^**	87.74 (34.71) **^b^**	74.69 (31.51) **^c^**	15.73	<0.001
Fiber (g) *	30.76 (51.48) **^a^**	31.28 (14.26) **^a^**	11.33 (8.29) **^b^**	20.08	<0.001
**Micro Nutrients**					
Thiamin (mg) Vit B1 *	1.52 (4.45) **^a^**	1.44 (0.79) **^a^**	0.58 (0.37) **^b^**	5.92	<0.001
Riboflavin (mg): Vit B2 *	1.90 (1.33) **^a^**	2.00 (0.88) **^a^**	0.73 (0.50) **^b^**	86.99	<0.001
Niacin equivalants (mg): Vit B3 *	39.27 (93.51) **^a^**	35.74 (21.72) **^a^**	15.35 (9.83) **^b^**	8.30	<0.001
Vit.C (mg) *	107.72 (60.46) **^a^**	**128.08 (71.58) **^b^****	40.19 (40.54) **^c^**	111.98	<0.001
Vit.D (mg) *	1.95 (1.29) **^a^**	1.72 (1.29) **^b^**	0.82 (0.89) **^c^**	49.73	<0.001
Vit.E (mg) *	13.60 (52.22) **^a^**	12.75 (8.11) **^a^**	4.55 (3.03) **^b^**	4.04	0.02
Folate-total (mg) *	611.25 (1,093.64) **^a^**	643.05 (319.52) **^a^**	201.58 (156.92) **^b^**	20.76	<0.001
Total Vit.A (mg) *	1,409.72 (1,651.87) **^a^**	1,570.64 (1,711.38) **^a^**	485.26 (641.23) **^b^**	28.51	<0.001
Retinol (mg) *	590.16 (1,451.00) **^a^**	589.26 (1,485.54) **^a^**	227.88 (535.00)	4.74	0.01
Beta carotene (mg) *	4,919.99 (3,157.51) **^a^**	**5,891.13 (3,405.77) **^b^****	1,545.10 (1,656.08) **^c^**	113.50	<0.001
Sodium (mg) *	965.47 (320.90) **^a^**	**1,017.21 (333.45) **^b^****	585.46 (256.18) **^c^**	111.34	<0.001
Potassium (mg) *	3,608.86 (3,712.56) **^a^**	3,785.69 (1,630.27) **^a^**	1,297.78 (887.17) **^b^**	52.21	<0.001
Magnesium (mg) *	**491.50 (1,197.23) **^a^****	473.85 (229.43) **^a^**	172.15 (108.54) **^b^**	9.70	<0.001
Calcium (mg) *	1,026.06 (688.00) **^a^**	1,006.13 (519.94) **^a^**	344.85 (320.50) **^b^**	91.58	<0.001
Phosphorus (mg) *	1,461.29 (2,644.43) **^a^**	1,337.43 (662.41) **^a^**	562.73 (350.45) **^b^**	14.58	<0.001
Iron (mg) *	18.93 (18.80) **^a^**	20.09 (8.64) **^a^**	6.90 (4.88) **^b^**	55.49	<0.001
Zinc (mg) *	11.18 (21.52) **^a^**	10.45 (4.98) **^a^**	4.48 (2.55) **^b^**	12.54	<0.001
Iodine (mg) *	91.11 (30.78) **^a^**	99.89 (35.01) **^b^**	40.87 (21.87) **^c^**	208.88	<0.001
**% Energy and Fat**					
Percent of total energy from protein, % *	24% (4.50%) **^a^**	23.26 (4.75) **^a^**	18.88 (8.68) **^b^**	48.71	<0.001
Percent of total energy from Fat. % *	36.27 (7.61) **^a^**	**38.51 (6.58) **^b^****	28.03 (12.95) **^c^**	87.96	<0.001
Percent of total energy from Saturated Fat, % *	8.67 (2.09) **^a^**	8.49 (1.92) **^a^**	6.07 (3.44) **^b^**	79.49	<0.001
Percent of total energy from Carbohydrate, % *	35.36 (9.35) **^b^**	33.37 (8.45) **^a^**	**49.75 (20.24) **^c^****	118.37	<0.001
Percent of total energy from Fiber, % *	3.67 (1.12) **^a^**	4.02 (1.16) **^a^**	2.68 (1.53) **^b^**	67.83	<0.001
Percent of total energy from Other sources, % *	0.90 (0.39) **^a^**	0.85 (0.35) **^a^**	0.66 (0.50) **^b^**	20.74	<0.001
Percent of total Fat as Mono Unsaturated Fats, % *	44.91 (4.65) **^a^**	47.47 (5.07) **^b^**	**48.87 (7.24) **^c^****	40.99	<0.001
Percent of total Fat as Poly Unsaturated Fats, % *	28.56 (6.96) **^a^**	28.32 (5.98) **^a^**	29.43 (9.28)	1.38	0.25
Percent of total Fat as saturated Fats, % *	26.38 (7.58) **^c^**	24.20 (6.33) **^b^**	21.71 (11.39) **^a^**	21.80	<0.001

Notes: * There are significant difference among different patterns using general linear model with *p* value less than 0.05, after adjustment for gender, age, by using general linear model factorial analysis. **^a,b,c^** values with different superscripts in the same row were significantly different by Duncan’s multiple range test at *p* value less than 0.01. Figures in bold show highest intake level.

Western dietary pattern had significantly higher percentage of total energy from fat, fibre, and higher percentage of total fat from mono unsaturated fat than both healthy dietary and balanced dietary pattern. 

### Chronic Disease Related Risk Factors

As indicated in Table 5, adults with Western dietary pattern had higher systolic and diastolic blood pressure levels, compared with their counterparts with healthy and balanced dietary pattern, and this has reached statistical significance in diastolic blood pressure. The level of triglycerides among adults with a Western dietary pattern was significantly higher than those with healthy dietary or balanced dietary patterns (1.797 ± 1.37 *vs.* 1.40 ± 0.07 and 1.07 ± 0.09*, p* < 0.001). Adults with the Western dietary and balanced pattern had much higher LDL levels than those with a healthy dietary pattern (2.86 ± 0.83 mmol/L and 2.86 ± 1.68 mmol/L, *vs.* 2.68 ± 0.94 mmol/L, *p* = 0.05). Adults with a Western dietary pattern had larger waist sizes than those with healthy dietary and balanced dietary patterns (Western dietary *vs.* healthy dietary pattern: 86.37 ± 12.74 *vs.* 80.16 ± 10.8, *p* < 0.001; Western dietary pattern *vs.* balanced dietary pattern: 86.37 ± 12.74 *vs.* 78.78 ± 0.76, *p* < 0.001). Adults with a Western dietary pattern also had statistically higher waist-hip ratios than those with a healthy dietary or balanced dietary pattern (Western dietary *vs.* healthy dietary: 0.86 ± 0.07 *vs.* 0.84 ± 0.07, *p* <0.001; Western dietary *vs.* balanced dietary: 0.86 ± 0.07 *vs.* 0.84 ± 0.05, *p* < 0.001). The level of Body Mass Index (BMI) among adults with a Western dietary pattern was significantly higher than among those with a healthy dietary pattern or balanced dietary pattern (Western dietary *vs.* healthy dietary: 25.68 ± 4.01 *vs.* 24.15 ± 3.38, *p* <0.001; Western dietary *vs.* balanced dietary: 25.68 ± 4.01 *vs.* 23.91 ± 3.13, *p* < 0.001).

**Table 5:** Association between dietary patterns and chronic disease related risk factors.

Biomarkers	Number of Participants	Healthy Dietary (n = 353)	Western Dietary (n = 343)	Balanced Dietary (n = 155)	*F* (2, 866)	*p*
**SBP**	353	130.81 (16.03)	132.93 (16.02)	130.38	2.02	0.13
**DBP**	353	82.76 (8.50) **^a^**	85.41 (9.57) **^b^**	81.84 **^a^**	**11.72**	**<0.001**
**Serum Cholesterol**	369	4.68 (2.69)	4.51 (1.15)	4.5723	0.67	0.51
**HDL (mmol/L)**	369	1.510 (0.51)	1.48 (0.47)	1.5010	0.30	0.74
**Triglycerides (mmol/L)**	369	1.40 (0.07) **^b^**	1.797 (1.37) **^a^**	1.07 (0.09) **^c^**	**10.62**	**<0.001**
**LDL (mmol/L)**	369	2.68 (0.94) **^a^**	2.86 (0.83) **^b^**	2.86 (1.68) **^b^**	2.96	**0.05**
**Fasting Glucose (mmol/L)**	369	5.53 (1.84)	5.36 (1.72)	5.58 (1.62)	1.16	0.32
**Serum Creatinine**	267	63.10 (13.79)	62.08 (12.51)	63.15 (10.49)	0.29	0.74
**eGFR**	262	91.37 (9.55)	92.43 (10.82)	91.96 (9.76)	0.49	0.64
**Waist (cm)**	345	80.16 (10.82) **^a^**	86.37 (12.74) **^b^**	78.78 (9.76) **^a^**	**33.14**	**<0.001**
**Hips (cm)**	345	95.91 (9.97) **^a^**	100.27 (10.69) **^b^**	94.05 (7.76) **^a^**	**25.29**	**<0.001**
**Waist Hip Ratio**	345	0.84 (0.07) **^a^**	0.86 (0.07) **^b^**	0.84 (0.05) **^c^**	**14.25**	**<0.001**
**BMI**	347	24.15 (3.38) **^a^**	25.68 (4.01) **^b^**	23.91 (3.13) **^a^**	**19.47**	**<0.001**

Notes: * There are significant difference among different patterns using general linear model with *p* value less than 0.05, after adjustment for gender, age, by using general linear model factorial analysis. **^a,b,c^** values with different superscripts in the same row were significantly different by Duncan’s multiple range test at *p* value less than 0.01. Figures in bold show statistical significance at *p* value less than 0.05.

Table 6 demonstrates prevalence and relative risks of obesity, hypertension, metabolic syndrome, dyslipidaemia and abnormal lipid profiles among adults in relation to three different dietary patterns. Obesity, central obesity, hypertension, abnormal triglycerides, and metabolic abnormality are more prevalent among adults with a Western dietary pattern, compared to those with a healthy or balanced dietary pattern. The relative risk ratio related to obesity among adults with a Western dietary pattern is 2.31 (95% CI: 1.15–5.88, *p* < 0.02), compared to adults with a healthy dietary pattern. When education, employment and income and exercise level were accounted for in the analysis, the relative risk ratio was reduced to 1.29 (95% CI: 0.97–6.32), but this finding was still statistically significant (*p* = 0.05). The risk ratio to have hypertension among adults with Western dietary pattern compared to those with a healthy dietary pattern was significant (Risk ratio: 2.05, 95% CI: 1.26–3.46, *p* = 0.007), when education, employment, income and exercise level were controlled in the analysis (Risk ratio: 1.95, 95% CI: 1.15–3.31, *p* = 0.01). The relative risk odds to have abnormal triglyceride level was higher among adults with a Western dietary pattern compared to those with a healthy dietary pattern in both the crude model (Risk ratio: 1.84, 95% CI: 1.08–3.42, *p* < 0.05) and adjusted model (Risk ratio: 1.80, 95% CI: 1.05–3.44, *p* = 0.05). Adults with a Western dietary pattern were more likely to have metabolic abnormality than those with a healthy dietary (Risk ratio: 1.58; 95% CI: 1.29–3.36, *p* = 0.01). This relationship remained significant (Risk ratio: 1.60; 95% CI: 1.09–2.68, *p* = 0.01) when confounding factors including education, employment, income, and exercise level were controlled in the analysis.

**Table 6:** Prevalence and relative risk (RR) of chronic disease related risk factors according to dietary patterns.

Risk Factors	Healthy Dietary (n = 353)	Western Dietary (n = 343)	Balanced Dietary (n = 155)
**Obesity**			
Prevalence	9.4%	21.2%	9.1%
Crude RR, *95% CI*	1	2.31 (1.15–5.88)	0.97 (0.39–2.41)
*p value, Z value*		*p < 0.02, Z = 2.31*	*p = 0.95, Z = −0.06*
Adjusted RR, *95% CI*	1	2.29 (0.97–6.32)	0.83 (0.28–2.50)
*p value, Z value*		*p = 0.05, Z = 2.20*	*p = 0.75, Z = −0.33*
**Central Obesity**			
Prevalence	41.2%	49.4%	38.0%
Crude Model, *95% CI*	1	1.06(0.57–1.81)	0.67 (0.34–1.32)
*p value. Z value*		*p = 0.84, Z = 0.21*	*p = 0.25, Z = −1.15*
Adjusted Model, *95% CI*	1	0.95 (0.50–1.81)	0.64 (0.32–1.29)
*p value, Z value*		*p = 0.88, Z= −0.15*	*p = 0.21, Z = −1.24*
**Hypertension (SBP/DBP: 140/90)**			
Prevalence	43.2%	53.7%	37.5%
Crude RR, *95% CI,*	1	2.05 (1.26–3.46)	1.06 (0.61–1.85)
*p value, Z value*		*p = 0.007, Z = 2.69*	*p = 0.83, Z = 0.21*
Adjusted RR, *95% CI,*	1	1.95 (1.15–3.31)	1.06 (0.61–1.85)
*p value, Z value*		*p = 0.01, Z = 2.50*	*p = 0.84, Z = 0.20*
**Abnormal Glucose (mmol/L)**			
Prevalence	48.9%	34.3%	63.2%
Crude RR, *95% CI*	1	0.25 (0.12–0.47)	0.80 (0.39–1.62), *p = 0.54*
*p value, Z value*		*p < 0.001. Z = −4.22*	
Adjusted RR, *95% CI*	1	23 (0.12–0.46)	0.73 (0.35–1.49)
*p value, Z value*		*p < 0.001, Z = −4.23*	*p = 0.38, Z = −0.87*
**Abnormal Triglycerides (mmol/L)**			
Prevalence	40.7%	55.5%	36.1%
Crude RR, *95% CI*	1	1.84 (1.08–3.42)	0.83 (0.42–1.63)
*p value, Z value*		*p < 0.05, Z = 1.92*	*p = 0.60, Z = −0.53*
Adjusted RR, *95% CI*	1	1.80 (1.05–3.44)	0.87 (0.43–1.79)
*p value, Z value*		*p = 0.05, Z = 1.90*	*p = 0.72, Z = −0.36*
**Cholesterol**			
Prevalence	31.1%	26.3%	33.3%
Crude RR, *95% CI*	1	0.84 (0.43–1.64)	1.18 (0.58–2.40)
*p value, Z value*		*p = 0.61, Z = −0.51*	*p = 0.65, Z = 0.45*
Adjusted RR, *95% CI*	1	0.81 (0.41–1.59),	1.17 (0.57–2.40)
*p value, Z value*		*p = 0.55, Z = −0.60*	*p = 0.67, Z = 0.43*
**Dyslipidemia**			
Prevalence	28.0%	30.1%	27.1%
Crude RR, *95% CI*	1	1.12 (0.57–2.23)	0.97 (0.46–2.03)
*p value, Z value*		*p = 0.73, Z=0.35*	*p = 0.94, Z = −0.08*
Adjusted RR, *95% CI*	1	1.10 (0.55–2.18)	0.92 (0.44–1.94)
*p value, Z value*		*p = 0.78, Z = 0.27*	*p = 0.84, Z = −0.20*
**Metabolic Syndrome**			
Prevalence	21.8%	30.0%	23.&%
Crude RR, *95% CI*	1	1.78 (1.29-3.36)	0.58 (0.27-1.21)
*p value, Z value*		*p = 0.01, Z = 3.01*	*p* = 0.15, Z = 1.44
Adjusted RR, *95% CI*	1	1.60 (1.09–2.68)	0.55 (0.26–1.16)
*p value, Z value*		*p = 0.02, Z = 2.59*	*p* = 0.12, Z = -1.57

Notes: In adjusted model education, employment, income and exercise level were controlled in the analysis. Figures in bold show statistical significance at *p* value less than 0.05.

## 5. Discussion

This study aimed to identify dietary patterns and their relationship with chronic disease related risk factors in Chinese older adults. Four food factors were identified, which included: a “traditional Chinese food” pattern, “Western dietary pattern”, “fast and processed food” pattern, and “animal organ”. Further analysis identified three dietary patterns, including a healthy dietary pattern, Western dietary pattern, and balanced dietary pattern. Healthy dietary pattern items predominantly include fish, pork, poultry, vegetables, fruits, nuts, soybean products, and rice in the food factor 1. The Western food pattern included red meat, flour grains, potatoes, light-coloured vegetables, peanuts, and fresh milk and the balanced food pattern included intakes foods across all four food factors. It was found that most Chinese adults predominantly choose healthy and Western dietary patterns, while adults with a balanced dietary pattern choose foods equally across four food factors but select lesser amounts of specific foods than those with healthy or Western dietary patterns. The “three dietary pattern” model is similar to the dietary patterns identified in previous studies [3].

Consistent with previous studies [21,22], the significant positive association between dietary patterns and prevalence of obesity suggests that a healthy dietary intake may decrease the chance of developing obesity, having high BP, high TG level, and metabolic abnormality. For example, it has been found that a healthy dietary pattern (*i.e.*, a higher intake of fish, poultry, rice, fruits and vegetables) is negatively associated with BMI, cardiovascular related biomarkers such as triglyceride [8], clinical level high blood pressure [23], and metabolic abnormality [3]. 

The healthy dietary pattern was inversely associated with a likelihood of having obesity (OR (95%CI): 0.51 (0.34–0.79), *p* = 0.002) compared to a Western dietary pattern and when qualification, employment, income and exercise level were accounted for in the analysis. A high intake of fruits and vegetables in the healthy diet may lead to lower metabolic, lipid protein, and vascular pressure levels. This may be a response to the high levels of antioxidants and vitamin E, vitamin B, and Vitamin D in fruit and vegetables, which are significantly related to decreased chance of obesity and cardiovascular related risks [3].

The potential of a Western dietary pattern to increase CVD risk factor incidents is consistent with findings in a Chinese study of dietary patterns. In this study, individuals who adopted a Western dietary pattern, which was characterized by higher intake of red meats of beef and lambs, moderate intake of pork and poultry, had an increased risk of having obesity, higher levels of systolic blood pressure (SBP), diastolic blood pressure (DBP), TG [3] and metabolic abnormality [3], compared to those with a healthy dietary pattern.

Our findings validate the correlation between food pattern and health outcome, and support previous studies, which found that the risk of ill health is associated with inappropriate nutrition in older adults [24,25]. Having a healthy dietary pattern and appropriate quantity of food intake, including fruits and vegetables and an appropriate amount of fish, poultry and milk products, can reduce the risks of having obesity, cardiovascular and metabolic risk factors such as obesity, high BP levels, TG, metabolic syndrome, all of which are related to chronic diseases such as diabetes and stroke, heart disease, hypertension. Consuming small amounts of a variety of foods and following national nutrition guidelines for people who have already had diabetes plays an important role in managing disease in Chinese older adults through planning and managing age-dependent nutrient intake.

This study had a high response rate and used a variety of statistical methods, including factor analysis, cluster analysis, and multiple logistic regression analysis to analyse Chinese dietary patterns, their relationship to chronic diseases and the FFQ’s sensitivity to differentiate between people with chronic diseases and those free from chronic conditions. There were two primary limitations of the study. First, data was collected from adults aged 50 and over with chronic disease in only two cities in China and we therefore cannot be sure that the results are generalizable to populations in other locations. Second, the study design is cross-sectional. As such, we cannot be conclusive about the causation of chronic diseases in relation to dietary pattern. Further research is required based on a prospective cohort or randomised control trial design to confirm the cause-effect relationships between dietary pattern and chronic diseases. 

## 6. Conclusions

In conclusion, this study provides evidence that the FFQ is a valid and reliable tool to assess the dietary patterns of individuals with chronic diseases in small- to medium-size urban and rural settings in China. It also validates the FFQ’s association with BP, BMI, WC, blood glucose, cholesterol, all of which underpin related metabolic conditions. Clinical diagnosis of chronic disease further confirmed the validation of the association of dietary pattern intake and chronic diseases. Understanding the need for and process of conducting a psychometric validity study is important for population health researchers. Population health practitioners are obligated to critically review measures because inadequate or inappropriate use of scales may result in methodologically significant problems. 
